# *N*-[4-Acetyl-5-methyl-5-(2-*p*-tolyl­prop­yl)-4,5-dihydro-1,3,4-thia­diazol-2-yl]acetamide

**DOI:** 10.1107/S1600536809000191

**Published:** 2009-01-10

**Authors:** Mohamed Tebaa, Noureddine Mazoir, Celia M. Maya, Bouhmaida Nouzha, Ahmed Benharref, Moha Berraho

**Affiliations:** a Laboratoire de Chimie Biomoléculaires, Substances Naturelles et Réactivité, Faculté des Sciences Semlalia, BP 2390 Bd My Abdellah, 40000 Marrakech, Morocco; b Instituto de Química Física Rocasolano, Consejo Superior de Investigaciones Científicas, Serrano, 119 28002 Madrid, Spain; c Laboratoire des Sciences des Matériaux, Département de Physique, Faculté des Sciences Semlalia, BP 2390 Bd My Abdellah, 40000 Marrakech, Morocco; d Laboratoire de Chimie Biomoléculaires, Substances Naturelles et Réactivité, Faculté des Sciences Semlalia, BP 2390 Bd My Abdellah, 40000 Marrakech, Morocco

## Abstract

The title heterocyclic compound, C_17_H_23_N_3_O_2_S, was synthesized from 4-(4-methyl­cyclo­hex-3-en­yl)pent-3-en-2-one, which was isolated from *Cedrus atlantica* essential oil. The thia­diazole ring adopts a flattened envelope conformation, with the flap *sp* ^3^-hybridized C atom lying 0.259 (1) Å out of the plane of the other four atoms. The screw-related mol­ecules are linked into chains along the *b* axis by inter­molecular N—H⋯O hydrogen bonds.

## Related literature

For 1,3,4-thia­diazole derivatives and their biological activity, see: Beatriz *et al.* (2002 ▶); Loughzail *et al.* (2009 ▶); Mazoir *et al.* (2008 ▶); Mohammed *et al.* (2008 ▶); Nakagawa *et al.* (1996 ▶); Sakthivel *et al.* (2008 ▶); Tehranchian *et al.* (2005 ▶); Wang *et al.* (1999 ▶, 2004 ▶). For puckering parameters, see: Cremer & Pople (1975 ▶).
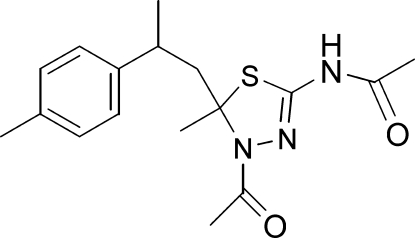


## Experimental

### 

#### Crystal data

C_17_H_23_N_3_O_2_S
*M* *_r_* = 333.44
Monoclinic, 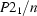

*a* = 9.3984 (2) Å
*b* = 11.0510 (2) Å
*c* = 16.6045 (3) Å
β = 90.442 (10)°
*V* = 1724.52 (6) Å^3^
*Z* = 4
Mo *K*α radiation
μ = 0.20 mm^−1^
*T* = 298 (2) K
0.5 × 0.4 × 0.3 mm

#### Data collection

Bruker X8 APEX CCD area-detector diffractometer
Absorption correction: none
52162 measured reflections
8286 independent reflections
7182 reflections with *I* > 2σ(*I*)
*R*_int_ = 0.019

#### Refinement

*R*[*F* ^2^ > 2σ(*F* ^2^)] = 0.035
*wR*(*F* ^2^) = 0.108
*S* = 1.03
8286 reflections
221 parameters
H atoms treated by a mixture of independent and constrained refinement
Δρ_max_ = 0.51 e Å^−3^
Δρ_min_ = −0.19 e Å^−3^

### 

Data collection: *APEX2* (Bruker, 2005 ▶); cell refinement: *SAINT-Plus* (Bruker, 2005 ▶); data reduction: *SAINT-Plus*; program(s) used to solve structure: *SHELXS97* (Sheldrick, 2008 ▶); program(s) used to refine structure: *SHELXL97* (Sheldrick, 2008 ▶); molecular graphics: *ORTEP-3* (Farrugia,1997 ▶) and *PLATON* (Spek, 2003 ▶); software used to prepare material for publication: *WinGX* (Farrugia, 1999 ▶).

## Supplementary Material

Crystal structure: contains datablocks I, global. DOI: 10.1107/S1600536809000191/ci2746sup1.cif

Structure factors: contains datablocks I. DOI: 10.1107/S1600536809000191/ci2746Isup2.hkl

Additional supplementary materials:  crystallographic information; 3D view; checkCIF report

## Figures and Tables

**Table 1:** Hydrogen-bond geometry (Å, °)

*D*—H⋯*A*	*D*—H	H⋯*A*	*D*⋯*A*	*D*—H⋯*A*
N1—H4⋯O2^i^	0.89 (1)	1.96 (1)	2.8391 (7)	169 (1)

Symmetry code: (i) 
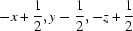
.

**Fig. 1.**
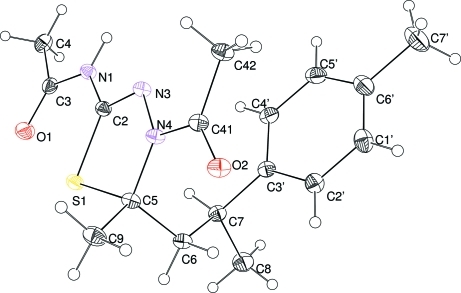
Molecular structure of the title compound with the atom-labelling scheme. Displacement ellipsoids are drawn at the 30% probability level. H atoms are represented as small spheres of arbitrary radii.

**Fig. 2.**
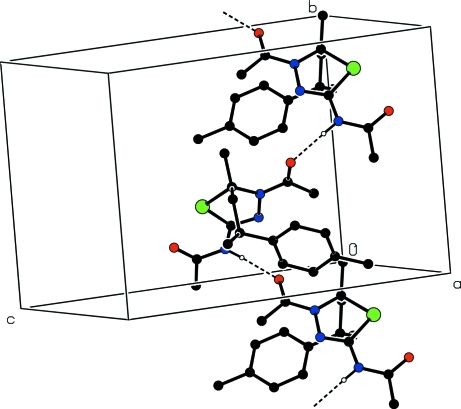
Partial packing view showing N—H···O hydrogen-bonded (dashed lines) chain running along the b axis. H atoms not involved in hydrogen bonding have been omitted for clarity.

## supplementary crystallographic information

### Comment

1,3,4-Thiadiazole derivatives (Sakthivel *et al.*, 2008) represent an interesting class of compounds possessing diverses activities: biological (Nakagawa *et al.*, 1996), fungicidal (Wang *et al.*, 1999, 2004) and bactericidal properties (Tehranchian *et al.*, 2005). The work of our research group focused on the phytochemical study of Moroccan plants and aimed to find out new compounds, which could be used as precursors or intermediates for the synthesis of high added value specimens (Mazoir *et al.*, 2008; Loughzail *et al.*,2009). In this way, we have investigated native Cedrus species rich on sesquiterpene derivatives. Thus a new compound was obtained through chemical modification of 4-(4-methylcyclohex-3-enyl)pent-3-en-2-one, which was isolated from Cedrus Atlantica essential oil. The aromatization of the above compound followed by condensation with thiosemicarbazide (Beatriz *et al.*, 2002; Mohammed *et al.*, 2008) ending with treatment of acetic anhydride in the presence of pyridine yielded a diasterioisomers in high stereoselectivity.

The molecular structure of the title compound is shown in Fig. 1. The thiadiazole ring adopts a flattened envelop conformation as indicated by Cremer & Pople (1975) puckering parameters Q = 0.1578 (6) Å and φ = 148.3 (2)°. Atom C5 deviates from the mean plane through other four atoms in the ring by 0.259 (1) Å.

In the crystal structure, molecules are linked into chains (Fig. 2) running along the *b* axis by intermolecular N—H···O hydrogen bonds (Table 1) involving the carbonyl and the acetamide groups.

### Experimental

A solution of 4-(4-methylcyclohex-3-enyl)pent-3-en-2-one (0.5 g, 2.8 mmol) and Pd/C (10%) was heated at 423 K for 12 h. The product obtained was treated with equimolecular quantity of thiosemicarbazide and several drops of HCl (cc) were added. The reaction mixture was heated at reflux in ethanol for 6 h and then evaporated under reduced pressure and the residue obtained was purified on silica gel column using hexane-ethyl acetate (96:4) as an eluent. 0.25 mmol of the thiosemicarbazone obtained was dissolved in 3 ml of pyridine and 3 ml of acetic anhydride. The mixture was heated on a water bath for 1.5 h. The resulting residue was concentrated *in vacuo* and chromatographied on silica gel column with hexane-ethyl acetate (92:8) as an eluent. Suitable crystals were obtained by evaporation of ethyl acetate solution at 277 K.

### Refinement

Atoms H4 and H7 were located in a difference map and refined freely (C7—H7 = 0.974 (11) Å and N1—H4 = 0.889 (13) Å). The remaining H atoms were fixed geometrically and treated as riding with C—H = 0.93 Å (aromatic), 0.96 Å (methyl), 0.97 Å (methylene), 0.98Å (methine) with *U*_iso_(H) = 1.2*U*_eq_(aromatic, methylene, methine) or *U*_iso_(H) = 1.5*U*_eq_(methyl). The highest residual density peak is located 0.62 Å from atom C2 and the deepest hole is located 0.39 Å from atom H70'.

### Crystal data

**Table:**

C_17_H_23_N_3_O_2_S	*F*(000) = 712
*M**_r_* = 333.44	*D*_x_ = 1.284 Mg m^−^^3^
Monoclinic, *P*2_1_/*n*	Mo *K*α radiation, λ = 0.71073 Å
Hall symbol: -P 2yn	Cell parameters from 31976 reflections
*a* = 9.3984 (2) Å	θ = 2.2–36.5°
*b* = 11.0510 (2) Å	µ = 0.20 mm^−^^1^
*c* = 16.6045 (3) Å	*T* = 298 K
β = 90.442 (10)°	Prism, colourless
*V* = 1724.52 (6) Å^3^	0.5 × 0.4 × 0.3 mm
*Z* = 4	

### Data collection

**Table:**

Bruker X8 APEX CCD area-detector diffractometer	7182 reflections with *I* > 2σ(*I*)
Radiation source: fine-focus sealed tube	*R*_int_ = 0.019
graphite	θ_max_ = 36.8°, θ_min_ = 2.2°
φ and ω scans	*h* = −14→15
52162 measured reflections	*k* = −18→17
8286 independent reflections	*l* = −27→27

### Refinement

**Table:**

Refinement on *F*^2^	Primary atom site location: structure-invariant direct methods
Least-squares matrix: full	Secondary atom site location: difference Fourier map
*R*[*F*^2^ > 2σ(*F*^2^)] = 0.035	Hydrogen site location: inferred from neighbouring sites
*wR*(*F*^2^) = 0.108	H atoms treated by a mixture of independent and constrained refinement
*S* = 1.03	*w* = 1/[σ^2^(*F*_o_^2^) + (0.0594*P*)^2^ + 0.2773*P*] where *P* = (*F*_o_^2^ + 2*F*_c_^2^)/3
8286 reflections	(Δ/σ)_max_ = 0.002
221 parameters	Δρ_max_ = 0.51 e Å^−^^3^
0 restraints	Δρ_min_ = −0.19 e Å^−^^3^

### Fractional atomic coordinates and isotropic or equivalent isotropic displacement parameters (Å^2^)

**Table:**

	*x*	*y*	*z*	*U*_iso_*/*U*_eq_	
C1'	−0.18648 (9)	0.69929 (8)	0.22865 (5)	0.03405 (16)	
H1'	−0.2631	0.7423	0.2493	0.041*	
C2'	−0.12028 (8)	0.74007 (7)	0.15872 (5)	0.02883 (13)	
H2'	−0.1553	0.8087	0.1329	0.035*	
C2	0.40705 (7)	0.72005 (6)	0.13520 (4)	0.02074 (10)	
C3'	−0.00246 (7)	0.68003 (6)	0.12659 (4)	0.02299 (11)	
C3	0.58917 (7)	0.58558 (7)	0.08145 (4)	0.02531 (12)	
C4	0.67040 (8)	0.47195 (8)	0.09881 (5)	0.03082 (14)	
H40	0.6397	0.4094	0.0625	0.046*	
H41	0.6534	0.4471	0.1533	0.046*	
H42	0.7702	0.4865	0.0917	0.046*	
C4'	0.04426 (8)	0.57608 (7)	0.16569 (5)	0.02818 (13)	
H4'	0.1221	0.5340	0.1458	0.034*	
C5	0.24444 (7)	0.89236 (6)	0.09425 (4)	0.02261 (11)	
C5'	−0.02462 (10)	0.53384 (8)	0.23496 (5)	0.03469 (17)	
H5'	0.0073	0.4631	0.2595	0.042*	
C6	0.10835 (8)	0.85717 (6)	0.04946 (4)	0.02444 (12)	
H61	0.1172	0.8834	−0.0060	0.029*	
H62	0.0302	0.9021	0.0728	0.029*	
C6'	−0.13955 (10)	0.59549 (9)	0.26777 (5)	0.03553 (17)	
C7'	−0.21196 (14)	0.55330 (13)	0.34392 (6)	0.0557 (3)	
H70'	−0.1661	0.4813	0.3634	0.083*	
H71'	−0.3103	0.5363	0.3325	0.083*	
H72'	−0.2054	0.6155	0.3841	0.083*	
C7	0.06784 (7)	0.72237 (6)	0.04914 (4)	0.02407 (11)	
C8	−0.02971 (10)	0.69513 (9)	−0.02232 (5)	0.03593 (17)	
H80	0.0164	0.7187	−0.0713	0.054*	
H81	−0.1169	0.7395	−0.0168	0.054*	
H82	−0.0501	0.6100	−0.0239	0.054*	
C9	0.27064 (10)	1.02844 (7)	0.08864 (5)	0.03188 (15)	
H90	0.3533	1.0493	0.1200	0.048*	
H91	0.1895	1.0711	0.1090	0.048*	
H92	0.2855	1.0505	0.0334	0.048*	
C41	0.15568 (7)	0.88983 (6)	0.23768 (4)	0.02220 (11)	
C42	0.16402 (9)	0.82781 (7)	0.31792 (4)	0.02797 (13)	
H420	0.1094	0.7544	0.3161	0.042*	
H421	0.1265	0.8802	0.3587	0.042*	
H422	0.2615	0.8091	0.3304	0.042*	
N1	0.49426 (6)	0.61997 (5)	0.14026 (3)	0.02274 (10)	
N3	0.32596 (6)	0.74595 (5)	0.19539 (3)	0.02162 (10)	
N4	0.24397 (6)	0.84788 (5)	0.17882 (3)	0.02167 (10)	
O1	0.60479 (7)	0.64374 (7)	0.01970 (4)	0.03731 (14)	
O2	0.07229 (6)	0.97409 (5)	0.22442 (3)	0.02734 (10)	
S1	0.400452 (18)	0.813333 (16)	0.050251 (10)	0.02473 (5)	
H4	0.4838 (13)	0.5774 (12)	0.1853 (8)	0.036 (3)*	
H7	0.1540 (12)	0.6750 (10)	0.0411 (7)	0.028 (3)*	

### Atomic displacement parameters (Å^2^)

**Table:**

	*U*^11^	*U*^22^	*U*^33^	*U*^12^	*U*^13^	*U*^23^
C1'	0.0346 (4)	0.0368 (4)	0.0309 (3)	−0.0069 (3)	0.0063 (3)	−0.0080 (3)
C2'	0.0302 (3)	0.0267 (3)	0.0297 (3)	0.0019 (2)	0.0030 (2)	−0.0024 (2)
C2	0.0225 (2)	0.0212 (2)	0.0185 (2)	−0.00085 (19)	−0.00014 (18)	0.00030 (19)
C3'	0.0255 (3)	0.0214 (3)	0.0220 (3)	0.0002 (2)	−0.0027 (2)	−0.00210 (19)
C3	0.0203 (2)	0.0334 (3)	0.0222 (3)	0.0008 (2)	0.00024 (19)	−0.0032 (2)
C4	0.0255 (3)	0.0331 (3)	0.0338 (3)	0.0052 (2)	−0.0005 (2)	−0.0081 (3)
C4'	0.0296 (3)	0.0246 (3)	0.0303 (3)	−0.0003 (2)	−0.0072 (2)	0.0011 (2)
C5	0.0293 (3)	0.0197 (2)	0.0188 (2)	0.0009 (2)	−0.0005 (2)	0.00177 (19)
C5'	0.0396 (4)	0.0325 (4)	0.0318 (3)	−0.0098 (3)	−0.0116 (3)	0.0077 (3)
C6	0.0289 (3)	0.0245 (3)	0.0199 (2)	0.0025 (2)	−0.0023 (2)	0.0024 (2)
C6'	0.0407 (4)	0.0420 (4)	0.0238 (3)	−0.0185 (3)	−0.0029 (3)	−0.0004 (3)
C7'	0.0633 (7)	0.0724 (8)	0.0313 (4)	−0.0341 (6)	0.0034 (4)	0.0054 (5)
C7	0.0264 (3)	0.0251 (3)	0.0207 (2)	0.0018 (2)	−0.0016 (2)	−0.0032 (2)
C8	0.0402 (4)	0.0437 (4)	0.0238 (3)	−0.0051 (3)	−0.0066 (3)	−0.0053 (3)
C9	0.0445 (4)	0.0202 (3)	0.0310 (3)	−0.0016 (3)	−0.0006 (3)	0.0036 (2)
C41	0.0276 (3)	0.0205 (2)	0.0185 (2)	0.0008 (2)	−0.00094 (19)	−0.00301 (19)
C42	0.0367 (3)	0.0281 (3)	0.0191 (3)	0.0049 (3)	0.0014 (2)	0.0006 (2)
N1	0.0240 (2)	0.0233 (2)	0.0209 (2)	0.00241 (18)	0.00268 (17)	0.00121 (18)
N3	0.0262 (2)	0.0202 (2)	0.0184 (2)	0.00288 (18)	0.00029 (17)	0.00064 (17)
N4	0.0281 (2)	0.0199 (2)	0.0171 (2)	0.00304 (18)	−0.00031 (17)	0.00020 (16)
O1	0.0321 (3)	0.0550 (4)	0.0249 (2)	0.0069 (3)	0.0068 (2)	0.0066 (2)
O2	0.0344 (3)	0.0233 (2)	0.0244 (2)	0.00714 (18)	−0.00082 (18)	−0.00255 (17)
S1	0.02737 (8)	0.02713 (9)	0.01972 (8)	0.00035 (5)	0.00253 (5)	0.00433 (5)

### Geometric parameters (Å, °)

**Table:**

C1'—C6'	1.3886 (14)	C6—C7	1.5375 (10)
C1'—C2'	1.3963 (12)	C6—H61	0.97
C1'—H1'	0.93	C6—H62	0.97
C2'—C3'	1.4001 (10)	C6'—C7'	1.5142 (13)
C2'—H2'	0.93	C7'—H70'	0.96
C2—N3	1.2936 (8)	C7'—H71'	0.96
C2—N1	1.3788 (9)	C7'—H72'	0.96
C2—S1	1.7478 (6)	C7—C8	1.5240 (10)
C3'—C4'	1.3891 (10)	C7—H7	0.974 (11)
C3'—C7	1.5239 (10)	C8—H80	0.96
C3—O1	1.2199 (9)	C8—H81	0.96
C3—N1	1.3810 (9)	C8—H82	0.96
C3—C4	1.4966 (11)	C9—H90	0.96
C4—H40	0.96	C9—H91	0.96
C4—H41	0.96	C9—H92	0.96
C4—H42	0.96	C41—O2	1.2357 (8)
C4'—C5'	1.4040 (12)	C41—N4	1.3679 (8)
C4'—H4'	0.93	C41—C42	1.4998 (10)
C5—N4	1.4878 (8)	C42—H420	0.96
C5—C6	1.5250 (10)	C42—H421	0.96
C5—C9	1.5267 (10)	C42—H422	0.96
C5—S1	1.8609 (7)	N1—H4	0.889 (13)
C5'—C6'	1.3916 (14)	N3—N4	1.3911 (8)
C5'—H5'	0.93		
			
C6'—C1'—C2'	120.90 (8)	C6'—C7'—H70'	109.5
C6'—C1'—H1'	119.5	C6'—C7'—H71'	109.5
C2'—C1'—H1'	119.5	H70'—C7'—H71'	109.5
C1'—C2'—C3'	121.52 (7)	C6'—C7'—H72'	109.5
C1'—C2'—H2'	119.2	H70'—C7'—H72'	109.5
C3'—C2'—H2'	119.2	H71'—C7'—H72'	109.5
N3—C2—N1	118.94 (6)	C3'—C7—C8	109.53 (6)
N3—C2—S1	118.40 (5)	C3'—C7—C6	113.80 (5)
N1—C2—S1	122.66 (5)	C8—C7—C6	109.97 (6)
C4'—C3'—C2'	117.51 (7)	C3'—C7—H7	108.5 (7)
C4'—C3'—C7	120.72 (6)	C8—C7—H7	106.4 (7)
C2'—C3'—C7	121.70 (6)	C6—C7—H7	108.3 (7)
O1—C3—N1	122.14 (7)	C7—C8—H80	109.5
O1—C3—C4	122.65 (7)	C7—C8—H81	109.5
N1—C3—C4	115.21 (6)	H80—C8—H81	109.5
C3—C4—H40	109.5	C7—C8—H82	109.5
C3—C4—H41	109.5	H80—C8—H82	109.5
H40—C4—H41	109.5	H81—C8—H82	109.5
C3—C4—H42	109.5	C5—C9—H90	109.5
H40—C4—H42	109.5	C5—C9—H91	109.5
H41—C4—H42	109.5	H90—C9—H91	109.5
C3'—C4'—C5'	120.78 (8)	C5—C9—H92	109.5
C3'—C4'—H4'	119.6	H90—C9—H92	109.5
C5'—C4'—H4'	119.6	H91—C9—H92	109.5
N4—C5—C6	111.56 (5)	O2—C41—N4	121.03 (6)
N4—C5—C9	112.62 (6)	O2—C41—C42	122.10 (6)
C6—C5—C9	110.89 (6)	N4—C41—C42	116.87 (6)
N4—C5—S1	102.92 (4)	C41—C42—H420	109.5
C6—C5—S1	110.44 (5)	C41—C42—H421	109.5
C9—C5—S1	108.08 (5)	H420—C42—H421	109.5
C6'—C5'—C4'	121.47 (8)	C41—C42—H422	109.5
C6'—C5'—H5'	119.3	H420—C42—H422	109.5
C4'—C5'—H5'	119.3	H421—C42—H422	109.5
C5—C6—C7	117.10 (5)	C2—N1—C3	124.37 (6)
C5—C6—H61	108.0	C2—N1—H4	113.9 (8)
C7—C6—H61	108.0	C3—N1—H4	121.7 (8)
C5—C6—H62	108.0	C2—N3—N4	110.79 (5)
C7—C6—H62	108.0	C41—N4—N3	118.12 (5)
H61—C6—H62	107.3	C41—N4—C5	124.65 (5)
C1'—C6'—C5'	117.78 (7)	N3—N4—C5	116.65 (5)
C1'—C6'—C7'	120.12 (10)	C2—S1—C5	89.03 (3)
C5'—C6'—C7'	122.10 (10)		

### Hydrogen-bond geometry (Å, °)

**Table:**

*D*—H···*A*	*D*—H	H···*A*	*D*···*A*	*D*—H···*A*
N1—H4···O2^i^	0.89 (1)	1.96 (1)	2.8391 (7)	169 (1)

Symmetry codes: (i) −*x*+1/2, *y*−1/2, −*z*+1/2.

## References

[bb1] Beatriz, N. B., Albertina, G. M., Miriam, M. A., Angel, A. L., Graciela, Y. M. & Norma, B. D. (2002). Arkivok, X, 14-23.

[bb2] Bruker (2005). *APEX2* and *SAINT-Plus* Bruker AXS Inc., Madison, Wisconsin, USA.

[bb3] Cremer, D. & Pople, J. A. (1975). *J. Am. Chem. Soc.* **97**, 1354–1358.

[bb4] Farrugia, L. J. (1997). *J. Appl. Cryst.* **30**, 565.

[bb5] Farrugia, L. J. (1999). *J. Appl. Cryst.* **32**, 837–838.

[bb6] Loughzail, M., Mazoir, N., Maya, C. M., Berraho, M., Benharref, A. & Bouhmaida, N. (2009). *Acta Cryst.* E**65**, o4. 10.1107/S1600536808039998 PMC2967856 21581682

[bb7] Mazoir, N., Benharref, A., Bailén, M., Reina, M. & González-Coloma, A. (2008). *Phytochemistry*, **69**, 1328–1338. 10.1016/j.phytochem.2008.01.004 18304594

[bb8] Mohammed, T., Mazoir, N., Daran, J.-C., Berraho, M. & Benharref, A. (2008). *Acta Cryst.* E**64**, o610–o611. 10.1107/S1600536808004728 PMC2960842 21201946

[bb9] Nakagawa, Y., Nishimura, K., Izumi, K., Kinoshita, K., Kimura, T. & Kurihara, N. (1996). *J. Pestic. Sci.* **21**, 195–201.

[bb10] Sakthivel, P., Joseph, P. S., Muthiah, P. T., Sethusankar, K. & Thennarasu, S. (2008). *Acta Cryst.* E**64**, o216. 10.1107/S1600536807064823 PMC2915277 21200782

[bb11] Sheldrick, G. M. (2008). *Acta Cryst.* A**64**, 112–122. 10.1107/S0108767307043930 18156677

[bb12] Spek, A. L. (2003). *J. Appl. Cryst.* **36**, 7–13.

[bb13] Tehranchian, S., Akbarzadeh, T., Fazeli, R. M., Jamifar, H. & Shafiee, A. (2005). *Bioorg. Med. Chem. Lett.* **15**, 1023–1025. 10.1016/j.bmcl.2004.12.039 15686905

[bb14] Wang, Y.-G., Cao, L., Yang, J., Ye, W.-F., Zhou, Q.-C. & Lu, B.-X. (1999). *Chem. J. Chin. Univ.* **20**, 1903–1905.

[bb15] Wang, Y.-G., Wang, Z. Y., Zhao, X. Y. & Song, X. J. (2004). *Chin. J. Org. Chem.* **24**, 1606–1609.

